# Indications for Keratoplasty in King Abdul-Aziz University Hospital: Five Years of Experience

**DOI:** 10.21315/mjms-03-2025-170

**Published:** 2025-08-30

**Authors:** Saeed Saleh Alqahtani

**Affiliations:** Department of Surgery, College of Medicine, Najran University, Najran, Kingdom of Saudi Arabia

**Keywords:** corneal transplantation, deep anterior lamellar keratoplasty, Descemet stripping automated endothelial keratoplasty, lamellar keratoplasty, penetrating keratoplasty

## Abstract

**Background:**

This study aimed to describe the indications and surgical techniques for corneal transplantation performed at a tertiary hospital over the past five years.

**Methods:**

A retrospective cross-sectional chart review was conducted of medical records for keratoplasty cases admitted to the hospital during this period. For each patient, data were collected on demographic characteristics, clinical indications for keratoplasty, associated ocular conditions, the surgical technique used, graft size, postoperative outcomes, suture removal time, and complication rates.

**Results:**

A total of 132 patients (159 keratoplasties) were included. The leading indications for keratoplasty were keratoconus (74/159, 46.5%), corneal decompensation including pseudophakic bullous keratopathy (37/159, 23.3%), and microbial keratitis (10/159, 6.3%). Penetrating keratoplasty (PKP) was the most commonly performed surgical technique (84/159, 53%), followed by Descemet’s stripping automated endothelial keratoplasty (DSAEK, 35/159, 22%). Postoperatively, mean intraocular pressure remained stable (approximately 16–17 mmHg) throughout follow-up visits. By two months, corneal clarity was achieved in nearly 90% of cases, with gradual improvement in visual acuity up to three months.

**Conclusions:**

The findings highlight key indications and evolving surgical techniques in corneal transplantation, underscoring the importance of early diagnosis and adoption of less invasive procedures to improve patient outcomes. These insights support targeted clinical strategies and resource planning to optimise corneal transplant care.

## Introduction

Corneal transplantation, also known as keratoplasty, is one of the most commonly performed types of transplantation worldwide, with a high success rate (1). The first successful corneal transplantation was performed by Eduard Zirm in 1905 (2). For nearly a century, surgical techniques remained relatively unchanged, with only minor modifications such as the introduction of microscopes, sutures, and medications like antibiotics and corticosteroids. However, since the first decade of the 21st century, corneal transplantation has undergone significant advancements with more selective and refined replacement procedures (3). This evolution has been driven by an improved understanding of corneal anatomy and the development of better surgical tools and microscopes. Consequently, keratoplasty has progressed from full-thickness corneal replacement to selective transplantation of specific corneal layers (1, 4).

The cornea’s unique properties facilitate its preservation and successful transplantation. Eye banks play a crucial role in the collection, storage, and distribution of corneal tissue. Recent advances in corneal transplantation techniques have revolutionised the field. A major milestone was achieved in 2006 when Melles et al. introduced Descemet’s membrane endothelial keratoplasty (DMEK), a novel procedure that allows transplantation of isolated Descemet’s membrane via a self-sealing tunnel incision (3).

Prior to DMEK, various lamellar keratoplasty techniques—including Descemet stripping endothelial keratoplasty (DSEK) and Descemet stripping automated endothelial keratoplasty (DSAEK)—were developed (5, 6). These less invasive techniques offer several advantages over penetrating keratoplasty (PKP), including lower rejection rates and quicker visual recovery (3, 7, 8). In 2006, Darlington et al. conducted a retrospective analysis using data from the Eye Bank Association of America to assess trends in PKP procedures in the United States (9). Their analysis, covering 1980 to 2004, showed that over 95% of corneal tissues were used for PKP, with the most common indications being pseudophakic bullous keratopathy (PBK), followed by keratoconus and Fuchs’ dystrophy.

Similarly, a retrospective analysis of data from four centres in the Eastern Province of Saudi Arabia revealed that the leading indications for keratoplasty were keratoconus (53.1%), followed by bullous keratopathy, corneal scarring, regrafts, and stromal dystrophies (10). The aim of this study was to describe the indications and surgical techniques for corneal transplantation performed at King Abdul-Aziz University Hospital over the past five years.

## Methods

This study was approved by the Research Ethics Committee of King Abdul-Aziz University Hospital (Ref. No. 17/0003/IRB). Manuscript preparation adhered to the Strengthening the Reporting of Observational Studies in Epidemiology (STROBE) guidelines (11) and was conducted in accordance with the ethical principles outlined in the Declaration of Helsinki (12).

### Study Design, Setting and Duration

A retrospective cross-sectional chart review was conducted of all patients who underwent corneal transplantation at King Abdul-Aziz University Hospital, Jeddah, Saudi Arabia, between 1 January 2020 and 31 December 2024.

### Eligibility Criteria

All patients who underwent any form of keratoplasty during the specified study period were eligible for inclusion, irrespective of age, gender, nationality, or laterality of the procedure. Procedures included penetrating keratoplasty (PKP), deep anterior lamellar keratoplasty (DALK) and DSAEK.

Patients were excluded if their medical records were incomplete or if key clinical data relevant to study endpoints (e.g., indication for surgery, surgical technique, postoperative outcomes) were missing. All surgeries were performed by experienced corneal surgeons following standardised institutional protocols.

### Data Collection

Data extraction was conducted retrospectively through a systematic review of electronic medical records within the hospital database for all eligible patients. Extraction was performed over a defined timeframe following ethical approval, using a standardised data collection form to ensure thorough and consistent capture of demographic, clinical, surgical, and postoperative outcome variables. Extracted data included demographic characteristics (age, gender, nationality), clinical indications for keratoplasty, associated ocular comorbidities, surgical details (type of keratoplasty, graft size), and postoperative outcomes. Outcomes evaluated were intraocular pressure (IOP), visual acuity (VA) measured using the Logarithm of the Minimum Angle of Resolution (LogMAR) scale, corneal clarity, timing of suture removal, and complication rates. To minimise selection bias, all consecutive patients undergoing keratoplasty during the study period were included regardless of demographic or clinical factors. Data extraction was performed by an experienced data collector using a standardised form to reduce information bias. A subset of records was independently reviewed for accuracy; no discrepancies were detected during this quality check.

### Data Analysis

Statistical analyses were performed using IBM SPSS Statistics version 24.0 (IBM Corp., Armonk, NY, USA). The normality of continuous variables was assessed using the Kolmogorov-Smirnov test. Descriptive statistics for continuous variables are presented as means with standard deviations (SD) when normally distributed or medians with interquartile ranges (IQR) when data were non-normally distributed. Categorical variables are summarised as frequencies and percentages. Due to the descriptive nature of this study, no inferential statistical comparisons were performed. Missing data were excluded from analyses on a case-by-case basis. A *P*-value less than 0.05 was considered statistically significant for any descriptive summaries.

## Results

### Patient Demographics and Baseline Characteristics

Over the past five years, a total of 159 corneal transplantations were performed in 132 patients, with a mean age of 46.5 years (SD = 22.1). A male predominance was observed, with 69 males (52.3%). Of the included patients, 125 (94.7%) were Saudi. Baseline characteristics are summarised in Table 1. A complete list of indications is also provided in Table 1.

### Types of Keratoplasty and Associated Ocular Conditions

The most common indications for keratoplasty were keratoconus (46.5%, *n* = 74), corneal decompensation (including PBK, 23.3%, *n* = 37), and microbial keratitis (6.3%, *n* = 10). Figure S1 shows yearly keratoplasty indications. Keratoconus was the most common indication (39.4%–53.3%), followed by corneal decompensation (21.2%–25.8%) and microbial keratitis (3.2%–9.1%). Other causes accounted for 20%–27.3%, with stable trends over the five years.

**Figure 1:**
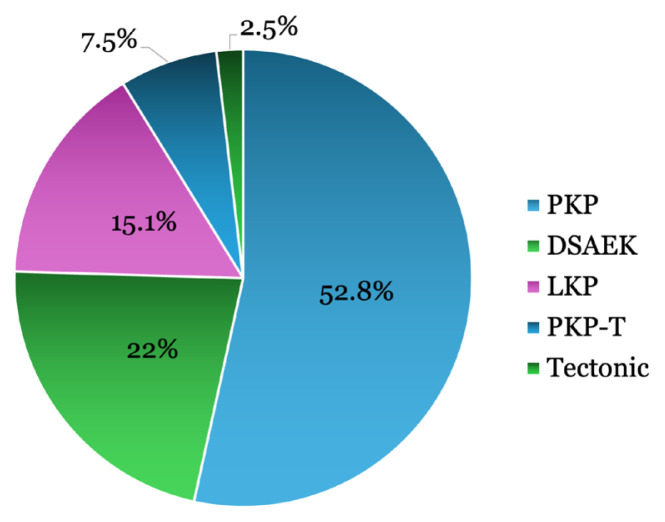
The most commonly performed keratoplasty procedures in King Abdul-Aziz University Hospital during the past 5 years PKP = penetrating keratoplasty; DSAEK = Descemet’s stripping automated endothelial keratoplasty; LKP = lamellar keratoplasty; t-PKP = penetrating keratoplasty with cataract extraction and intraocular lens implantation

The most frequently performed types of corneal transplantation were penetrating keratoplasty (PKP, 52.8%, *n* = 84), followed by DSAEK (22%, *n* = 35) and lamellar keratoplasty (LKP, 15.1%, *n* = 24), as shown in Figure 1. Less common procedures included PKP combined with cataract extraction and intraocular lens implantation (t-PKP) and tectonic PKP. Figure S2 shows the yearly distribution of keratoplasty techniques. The proportion of PKP decreased from 71.0% to 33.3%, while DSAEK and LKP increased. Other techniques remained minor throughout the study period.

**Figure 2:**
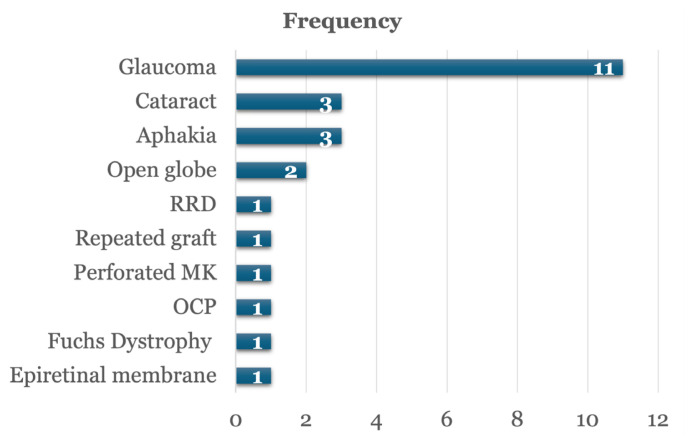
The associated ocular diseases RRD = rhegmatogenous retinal detachment; MK = microbial keratitis; OCP = Ocular Cicatricial Pemphigoid

Glaucoma was the most frequently reported associated ocular disease, present in 6.9% (*n* = 11) of patients, followed by aphakia and cataract in 1.9% (*n* = 3) each (Figure 2). The mean graft size used was 8.0 mm (SD = 1.0).

### Preoperative and Postoperative Outcomes (Table 2)

The preoperative and postoperative outcomes are presented in Table 2.

### Preoperative

The median preoperative VA was 2.0 (IQR = 1.3–2.0), approximately 20/400 to count fingers, and the mean preoperative IOP was 15.4 mmHg (SD = 5.7).

### One-month Follow-up

At one month postoperatively, the mean IOP was 17.4 mmHg (SD = 6.0). Among patients who underwent DSAEK (*n* = 35), 14 eyes had successful graft attachment, while one eye experienced graft detachment. Seven patients (4.7%) experienced complications, including two failed grafts, two cases of graft decentration, one Descemet’s membrane detachment, one case of endophthalmitis, and one case of persistent epithelial defect.

### Two-month Follow-up (Intermediate Time Point)

At two months, the mean VA improved to 1.0 (SD = 0.8), and the mean IOP was 16.3 mmHg (SD = 5.2). Corneal attachment was noted in 37.1% (*n* = 13) of patients, with full attachment in 8.6% (*n* = 3). Corneal clarity was achieved in 89.6% (*n* = 86). Concurrent medications included ofloxacin (2.5%, *n* = 4), corticosteroid (loteprednol, 0.6%, *n* = 1), and antiviral (acyclovir, 0.6%, *n* = 1).

### Three-month Follow-up

At three months, mean VA further improved to 0.7 (SD = 0.7), and mean IOP remained stable at 16.3 mmHg (SD = 4.2). The mean time to suture removal was 17.3 months (SD = 5.7).

## Discussion

This study investigated changes in surgical techniques and leading indications for corneal transplantation in 132 patients (159 keratoplasty procedures). The median age of patients was 40 years, with 69 males (52.3%). The most common indications for keratoplasty were keratoconus, followed by corneal decompensation (including PBK) and microbial keratitis. These findings align with previous studies, including that of Al-Arfai et al. (10), who analysed keratoplasty indications in Saudi Arabia from 2008 to 2013. Their study identified five leading indications—keratoconus, bullous keratopathy, corneal scarring, regrafts, and stromal dystrophies—which accounted for 92% of corneal transplants, with keratoconus being most common (53.1%).

Another large-scale study over 20 years showed a shift in indications. Early in the study, corneal scarring, PBK, corneal degeneration, and keratoconus were most common (52.0%, 13.5%, 10.0%, and 7.6%, respectively). Over the last five years, keratoconus has become the leading indication (40.2%), followed by corneal scarring (19.8%), failed grafts (11.3%), and corneal ulceration (10.2%). These changes were attributed to improvements in ophthalmic services, rapid socioeconomic development, and population growth (13–19).

The findings also correspond with recent international studies. Matthaei et al. (20) conducted a systematic review of 141 articles from 37 countries, identifying keratoconus as the leading indication for keratoplasty across Europe, Australia, the Middle East, Africa, and South America (22.8% to 33.2%). In contrast, North America and Asia reported higher frequencies of post-cataract surgery oedema and keratitis, respectively (20). Similarly, Bozkurt et al. (21) in Turkey reported keratoconus as the predominant indication (27.7%), followed by bullous keratopathy (23%), post-infectious corneal scars (13.5%), and regrafts (13.1%). Studies from New Zealand and Iran also identify keratoconus as the most common indication (22–24).

While keratoconus remains a leading cause of corneal transplantation globally, its prevalence varies with genetic, environmental, and geographic factors. Regions such as India and the Middle East report higher rates, potentially linked to environmental factors like excessive sun exposure and ultraviolet radiation (25–27). Bullous keratopathy continues as a common indication; however, advances in cataract surgery have reduced its prevalence, especially in developed countries (28).

Regarding surgical techniques, PKP was the most common procedure in this study, followed by DSAEK and lamellar keratoplasty (LKP). This pattern aligns with Al-Arfai et al. (10), who reported PKP as the most frequent procedure, followed by DALK and DSAEK. This study highlights a decline in PKP use in favour of lamellar keratoplasties. This trend mirrors findings by Chan et al. (29), who documented that lamellar keratoplasties accounted for 80% of cases, with PKP declining by 11%. Palma-Carvajal (30) reported that 61.75% of keratoplasties were lamellar procedures.

In this study, lamellar procedures represented less than half the number of PKP procedures, mainly because keratoconus was the primary indication for LKP, while alternative management options are becoming more common. PKP and DSAEK were preferred for bullous keratopathy, and PKP remained the main approach for infected corneal ulcers, failed grafts, and corneal scars.

Although this study was primarily descriptive without formal statistical analysis of temporal trends, data review suggests that the distribution of key indications and surgical techniques remained largely stable throughout the five-year period, likely reflecting consistent referral patterns and surgical practices at King Abdul-Aziz University Hospital. In contrast, South America reports a lower rate of lamellar endothelial techniques, possibly due to longer waiting lists and the expertise required for these advanced procedures (31).

Glaucoma was the most common associated ocular disease in this study, observed in 6.9% (*n* = 11) of patients, followed by aphakia and cataract (1.9% each). This aligns with Crawford et al. (22), who reported glaucoma in 12.8% of PKP recipients.

### Strengths and Limitations

This study has several strengths, including comprehensive data collection from all patients undergoing keratoplasty over a five-year period. Although the retrospective design limits causal inference and introduces potential biases such as selection and information bias, these were mitigated by including all consecutive patients and employing standardised data extraction performed by a single experienced investigator. A subset of records was independently reviewed for accuracy, and no discrepancies were detected during this quality check. Residual confounding cannot be excluded given the observational nature of the study, and this limitation is acknowledged.

While the study was not designed for formal temporal trend analysis, qualitative assessment suggests that indications and surgical techniques remained stable during the study period, reflecting consistent institutional practices. A future prospective study with larger datasets and formal trend analyses is planned to better characterise any changes over time. However, missing data on key clinical outcomes and the single-centre design limit the generalisability and completeness of findings. The absence of long-term follow-up restricts evaluation of graft survival and late complications. Although all surgeries were performed by expert corneal surgeons following standardised protocols, minor variations in surgical technique between surgeons may have influenced outcomes. These limitations underscore the need for future prospective, multicenter studies with standardised data collection and longer follow-up to confirm and expand upon these findings, including formal analyses of evolving trends.

## Conclusion

This study highlights the predominant indications for corneal transplantation at King Abdul-Aziz University Hospital (keratoconus, corneal decompensation including PBK, and microbial keratitis) and documents the increasing adoption of lamellar keratoplasty techniques, particularly DSAEK, alongside penetrating keratoplasty. Clinically, these findings emphasise the importance of early diagnosis and tailored management strategies for keratoconus and endothelial pathologies to potentially delay or reduce the need for transplantation. The shift towards endothelial keratoplasty supports the adoption of less invasive surgical approaches that may improve postoperative outcomes and reduce complication rates.

From a policy perspective, these results advocate for enhanced training and resource allocation to support advanced keratoplasty techniques in tertiary centres. Furthermore, developing standardised protocols for postoperative monitoring could optimise graft survival and visual rehabilitation. Future multicenter prospective studies with extended follow-up are warranted to validate these findings and guide evidence-based clinical guidelines, ultimately improving patient care and resource utilisation in corneal transplantation.

## Supplementary Materials

Figure S1 Yearly distribution of indications for keratoplasty over a 5-year period

Figure S2 Yearly trends in surgical techniques used for corneal transplantation

## Figures and Tables

**Table 1:** Demographic and clinical characteristics of the included patients.

**Age, years**	Median (Range): 40 (2–95)	
	Mean (SD): 46.5 (22.1)	

**Nationality, ** ***n*** ** (%)**	Saudi	125 (94.7)
	Others	7 (5.3)

**Gender, ** ***n*** ** (%)**	Male	69 (52.3)
	Female	63 (47.7)

**Laterality, ** ***n*** ** (%)**	Unilateral	109 (82.6)
	Bilateral	23 (17.4)

**Eye, ** ***n*** ** (%)**	Oculus Dexter (right eye)	78 (49.1)
	Oculus Sinister (left eye)	81 (50.9)

**Indications for keratoplasty procedures**	Keratoconus	74 (46.5)
	Corneal decompensation (including PBK)	37 (23.3)
	Microbial Keratitis	10 (6.3)
	Unknown causes of the corneal scars	8 (5.0)
	Macular Dystrophy	6 (3.8)
	Fuch’s Dystrophy	6 (3.8)
	Graft Rejection	4 (2.5)
	Perforated cornea with tissue loss	4 (2.5)
	Corneal Scar-Trauma	2 (1.3)
	Corneal Scar-Herpetic	2 (1.3)
	Corneal Scar-Glaucoma	2 (1.3)
	Open globe with tissue loss	2 (1.3)
	Corneal ulcer	1 (0.6)
	Band keratopathy	1 (0.6)

**Table 2:** IOP, VA, and clarity at various time points

Measurement	Mean (SD), [range]	Outcome Breakdown
IOP at 1 month (mmHg)^*^	17.4 (6.0), [8–36]	- **Attach**: 14/18 (77.78%) - **Detach**: 1/18 (5.56%) - **Full**: 3/18 (16.67%)
VA at 1 month (LogMAR)	1.0 (0.8), [0.04–3.0]	-
IOP at 2 months (mmHg)^**^	16.3 (5.2), [10–35]	- **Attach**: 13/16 (81.25%) - **Full**: 3/16 (18.75%)
VA at 2 months (LogMAR)	1.0 (0.8), [0.04–3.0]	-
Clarity at 2 months^***^		- **Clear**: 86/96 (89.58%) - **Not Clear**: 3/96 (3.13%) - **Opacity**: 2/96 (2.08%) - **Scar**: 2/96 (2.08%) - **Haze**: 1/96 (1.04%) - **Mild Oedema**: 1/96 (1.04%) - **Mild Haze**: 1/96 (1.04%)
IOP at 3 months (mmHg)	16.3 (4.2), [8.0–30.0]	-
VA at 3 months (LogMAR)	0.7 (0.7), [0.0–3.0]	-
Timing of suture removal (months)	17.3 (5.7), [4.0–24.0]	-

IOP = intraocular pressure; VA = visual acuity; LogMAR = Logarithm of the Minimum Angle of Resolution, a standard scale for measuring visual acuity; Data are missing for ^*^17; ^**^19; and ^***^63 keratoplasty procedures
