# Supplementary figures and images for: Indications for Keratoplasty in King Abdul-Aziz University Hospital: Five Years of Experience
Source: Malays J Med Sci. 2025 Aug 30;32(4):170–9. doi: 10.21315/mjms-03-2025-170 (PMC13132138; doi:10.21315/mjms-03-2025-170)

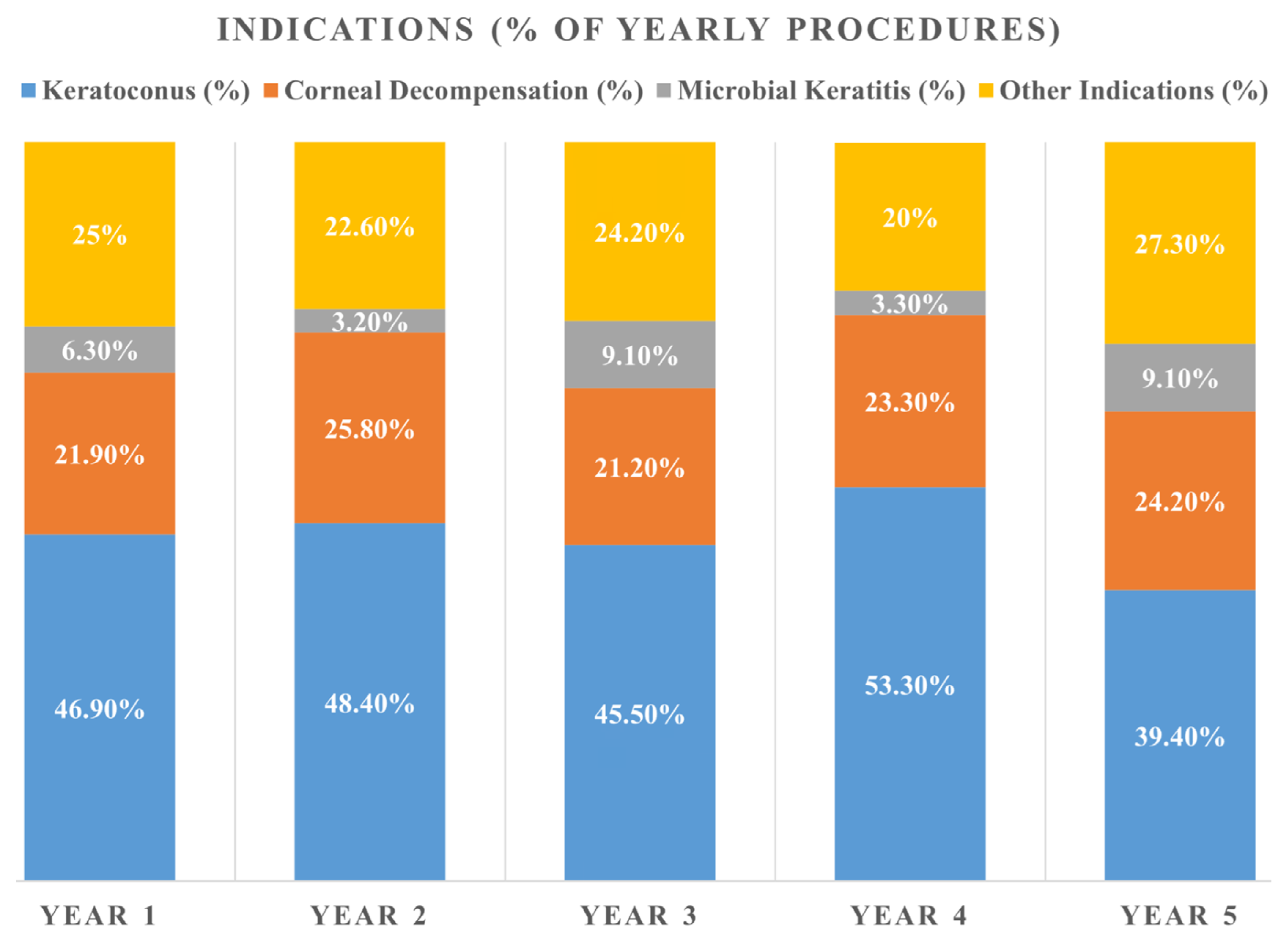

Supplement: Figure S1 — Yearly distribution of indications for keratoplasty over a 5-year period [file 11mjms3204_oas1.tif]

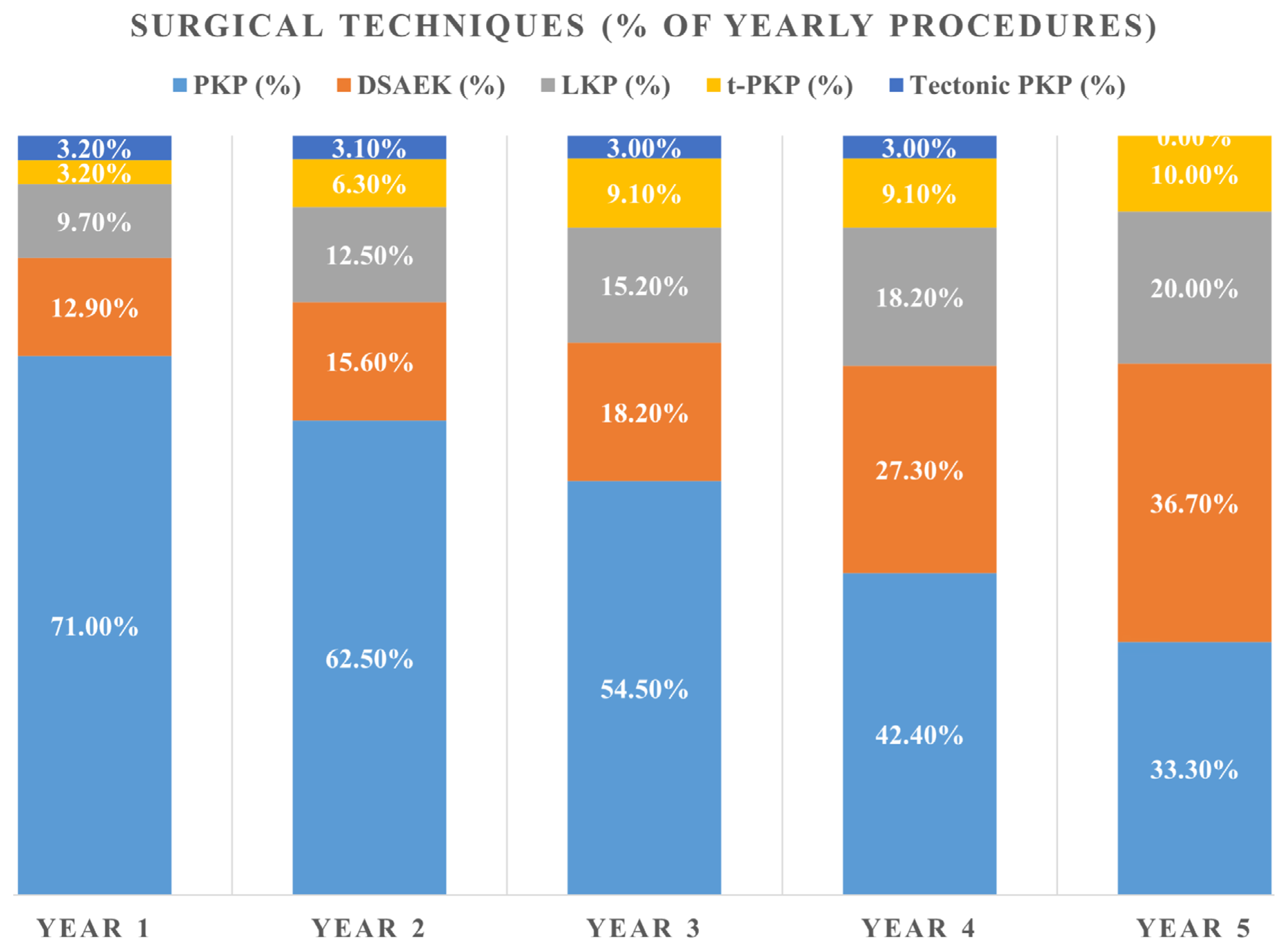

Supplement: Figure S2 — Yearly trends in surgical techniques used for corneal transplantation [file 11mjms3204_oas2.tif]
